# Thermo-optical characterization of fluorescent rhodamine B based temperature-sensitive nanosensors using a CMOS MEMS micro-hotplate^☆^

**DOI:** 10.1016/j.snb.2013.10.042

**Published:** 2014-03-01

**Authors:** Veeren M. Chauhan, Richard H. Hopper, Syed Z. Ali, Emma M. King, Florin Udrea, Chris H. Oxley, Jonathan W. Aylott

**Affiliations:** a Laboratory of Biophysics and Surface Analysis, School of Pharmacy, University of Nottingham, Boots Science Building, University Park, Nottingham NG7 2RD, UK; b Cambridge CMOS Sensors, Suite 820, 2nd Floor, St Andrews House, 59 St Andrews Street, Cambridge CB2 3BZ, UK; c Advanced Microscopy Unit, School of Biomedical Sciences, University of Nottingham, Queens Medical Centre, Nottingham NG7 2UH, UK; d Electrical Engineering Division, Engineering Department, University of Cambridge, 9 JJ Thomson Avenue, Cambridge CB3 0FA, UK; e Engineering, Faculty of Technology, De Montfort University, Queens Building, The Gateway, Leicester LE1 9BH, UK

**Keywords:** MEMS micro-hotplate, Fluorescent, Temperature-sensitive, Nanosensor, Rhodamine B, Silica sol–gel

## Abstract

A custom designed microelectromechanical systems (MEMS) micro-hotplate, capable of operating at high temperatures (up to 700 °C), was used to thermo-optically characterize fluorescent temperature-sensitive nanosensors. The nanosensors, 550 nm in diameter, are composed of temperature-sensitive rhodamine B (RhB) fluorophore which was conjugated to an inert silica sol–gel matrix. Temperature-sensitive nanosensors were dispersed and dried across the surface of the MEMS micro-hotplate, which was mounted in the slide holder of a fluorescence confocal microscope. Through electrical control of the MEMS micro-hotplate, temperature induced changes in fluorescence intensity of the nanosensors was measured over a wide temperature range. The fluorescence response of all nanosensors dispersed across the surface of the MEMS device was found to decrease in an exponential manner by 94%, when the temperature was increased from 25 °C to 145 °C. The fluorescence response of all dispersed nanosensors across the whole surface of the MEMS device and individual nanosensors, using line profile analysis, were not statistically different (*p* < 0.05). The MEMS device used for this study could prove to be a reliable, low cost, low power and high temperature micro-hotplate for the thermo-optical characterisation of sub-micron sized particles. The temperature-sensitive nanosensors could find potential application in the measurement of temperature in biological and micro-electrical systems.

## Highlights

• We report the development of a custom designed MEMS micro-hotplate capable of operating at high temperatures (up to 700 ̊C).
• The MEMS micro-hotplate can be directly mounted in a slide holder of a fluorescent confocal microscope.
• Temperature-sensitive nanosensors (550 nm diameter) were synthesised by covalently linking rhodamine B to a silica sol–gel matrix.
• Nanosensors were thermo-optically characterised using the MEMS device and confocal microscopy.
• The temperature dependent fluorescence response of the nanosensors was found to operate over wide range, up to 145 ̊C.

## 1 Introduction

Luminescent probes and sensors for temperature can provide accurate, reliable and reproducible optical characterization of the temperature [1] of biological systems [2] and microelectronic circuits [3], down to a single-molecule level [4]. A number of temperature dependent optical properties can be studied; such as changes in excitation and emission wavelength [5], fluorescence lifetime [6], emission intensity [7] and anisotropy [8], [9]. Thermo-optical characterization of the fluorophore involves modulation of the temperature, whilst one or more of these thermo-optical properties are monitored, using detection techniques such as fluorescence spectroscopy or microscopy.

Traditionally, hot stages, which consist of a large electrical heater element coupled to a computer based interface controller [10], have been used to thermally characterize temperature-sensitive samples. However, these conventional hot stages have a number of limitations. For example, microscopy equipment typically has to be specially adapted for thermo-optical characterization, as hot stages are traditionally cumbersome and cannot be mounted in the ubiquitous microscope slide holder [11]. Hot stages with a high temperature accuracy (<1 °C) are challenging to design and expensive to fabricate [12]. The heat generated from a hot stage's large thermal mass can cause heating of objective lenses, which may generate optical distortions and measurement artefacts [13]. Conventional electrical heating elements in these hot-stages consume large amounts of power, prohibiting mobile battery powered operation of the stage [13]. They have a slow thermal response (typically >1 °C/min) [10], which limits the rate at which thermo-optical measurements can be made.

Microelectromechanical systems (MEMS) micro-hotplates overcome some of the inherent challenges present with conventional hot stages:

1. These devices generally consist of a metallised heater element suspended on a thin silicon dioxide membrane which can be manufactured using a variety of semiconductor fabrication processes including chemical [14], plasma [15] and ion beam etching [16].
2. Due to the thermal isolation of the micro-hotplate, high temperature operation (up to ∼700 °C) can be achieved at very low power levels (<100 mW)
3. Their low thermal mass ensures relatively fast millisecond heating times (typically >50 °C/ms) [17], [18].
4. Careful electro-thermal design, with the help of computer modelling techniques, can ensure temperature uniformity (<1 °C) [18].
5. In addition, a temperature sensor (either a diode or a resistive sensor) can be embedded to provide feedback of the micro-hotplate's temperature [19].

MEMS fabrication has led to the development of a number of micron scaled hotplate devices, which have been used in a range of applications. For example: heaters for resistive/catalytic gas sensing [20], [21], [22], glass transition [23] and the thermal characterisation of nanomaterials [24].

Rhodamine B (RhB) is an example of a molecular probe, which has been extensively studied for its temperature dependent fluorescence response [25]. Its quantum yield, the ratio of photons emitted to photons absorbed, is highly temperature dependent [26], such that RhB's quantum yield decreases with an increase in temperature, which corresponds to a drop in emission intensity. However, the use of free fluorophores, like RhB, for temperature sensing is limited by; (1) the contamination of the surrounding material [27], due to adsorption on to surfaces, and (2) molecular interactions [28], [29], which can generate measurement artefacts due to non-temperature related changes in the fluorescence intensity. As a result there has been significant interest in the use of encapsulation matrices, such as, silica sol–gel [30], [31], poly(allylamine hydrochloride) [32] and poly(methyl methacrylate) [33], that exhibit superior properties when entrapping for temperature-sensitive fluorophores. Silica sol–gel matrices are; inert, homogenous and optically transparent. Silica is also photo and temperature stable, in some cases to 1300 °C [34], making silica sol–gel an ideal encapsulation matrix for dry quantitative spectrophotometric temperature measurements.

In this article we describe the development of a custom built complementary metal-oxide semiconductor (CMOS) MEMS micro-hotplate used to characterise the thermo-optical response of fluorescent temperature-sensitive nanosensors. The nanosensors, composed of RhB fluorophores conjugated to a silica sol–gel matrix, were dispersed and dried on the surface of the MEMS micro-hotplate, which was mounted on a conventional slide holder of a fluorescence confocal microscope. Temperature dependent changes in the fluorescence response of the nanosensors were evaluated by controlling the electrical power used to heat the MEMS micro-hotplate.

## 2 Materials

Tetraethyl orthosilicate (TEOS) and (3-aminopropyl) triethoxysilane (APTES) were obtained from Sigma-Aldrich (Gillingham, United Kingdom). Absolute ethanol was purchased from Fisher Scientific (Loughborough, United Kingdom). Ammonium hydroxide 30 wt% was obtained from Acros Organics (Loughborough, United Kingdom). Rhodamine B isothiocyanate (RhB-ITC) was purchased from MP Biomedicals (Cambridge, United Kingdom). Deionised water (18.2 MΩ) generated by Maxima HPLC grade USF Elga.

## 3 Experimental

### 3.1 Rhodamine B and APTES conjugation

RhB-ITC (5 mg, 0.01 mmol) was stirred in absolute ethanol (0.98 mL) for 10 min. APTES (20 μL, 0.09 mmol) was added in excess and stirred at 4 °C overnight. Aliquots of RhB-APTES conjugate solution were used for nanoparticle synthesis.

### 3.2 Temperature-sensitive nanosensor synthesis

A catalysis mixture was prepared by stirring ethanol (5.25 mL) and ammonium hydroxide (30% w/v, 4 mL) together for 10 min. In a separate container, a monomer solution was prepared by stirring TEOS (500 μL, 2.26 mmol) and RhB-APTES conjugate (250 μL from Rhb-APTES conjugate solution) together for 10 min. Nanosensor synthesis was initiated through the addition of monomer solution to the stirring of the catalysis solution at a controlled rate (75 μL/min, 10 min). The reaction was stirred for 1 h, producing an opaque nanoparticle suspension. Nanosensors were washed (10 times) through suspension in ethanol (30 mL) and centrifugation (6000 rpm, 10 min). Nanosensors were dried using vacuum filtration (200 nm pore membrane filter, Sartorius Stedim Biotech). The resultant solid was placed in a desiccator overnight to remove any remaining solvent.

### 3.3 Size characterization of nanosensors

#### 3.3.1 Scanning electron microscopy

Nanosensors were suspended in deionised water (20 μL, 0.25 mg/mL) and dried on an aluminium scanning electron microscopy stub (12.5 mm diameter, Agar Scientific). A Carl Zeiss EVO HD15 scanning electron microscope was used to image dry nanosensors (20 kV, 8 mm working distance). The measured diameters were validated using dynamic light scattering.

#### 3.3.2 Dynamic light scattering

Dynamic light scattering was performed using a Viscotek (802) system. The system is equipped with a 50 mW laser source (830 nm), operating at an angle of 90°. Nanosensors were suspended in deionised water (100 μL, 1 mg/mL). Measurements (10 runs, 25 °C) were made using a Hellma^®^ Analytics quartz cuvette (1.5 mm diameter). The mean hydrodynamic diameter of the particles was computed from the intensity of the scattered light using the OmniSize 3.0 software.

### 3.4 Deposition of temperature-sensitive particles on MEMS device

The temperature-sensitive nanosensors were suspended in deionised water (50 μL, 1 mg/mL). The suspension was deposited (1 μL) onto the MEMS micro-hotplate using a pipette and was allowed to evaporate to leave a thin film of dispersed nanosensors on the surface. An additional aliquot of nanosensors (1 μL) was also deposited on a glass coverslip (No. 1.5, 17 mm thick) so that they could be visualised as a high resolution image, using a fluorescence microscope and a high magnification oil immersion objective.

### 3.5 Imaging procedure

A Leica SP2 confocal fluorescence microscope coupled with a Leica HC PL FLUOTAR 50 × 0.8 NA (air) (pixel size 0.39 × 0.39 μm, pinhole 0.119 mm (1 Airy)) was used to image temperature-sensitive nanosensors. A Krypton 568 nm laser was used as excitation source. A photomultiplier tube (942 HV, 1.18 offset) was used to collect fluorescence between 580 nm and 625 nm. Acquired images were analysed using FIJI open source software. Background fluorescence was estimated by determining the fluorescence on MEMS devices coated without any particles and subtracted from images used for temperature analysis. Some warping of the membrane was found to occur due to thermal expansion, which can cause errors in the fluorescence intensity measurements especially when the sample becomes out of focus. This is more critical when using high magnification objectives with a short working distance. In order to correct for warping, the sample was refocused at each temperature point. Measurements of the focal point show that the movement of the membrane is of the order of 0.5 μm/°C (see supporting information, Fig. S1). Temperature induced fluorescence intensity changes for temperature-sensitive nanosensors were collected over the whole MEMS micro-hotplate area, and for individual dispersed nanosensors (*n* = 6) using line profile analysis, placed at the centre of a nanosensors surface intensity plot. Student's *t* test was used to identify significant differences between the full width half max (FWHM) of temperature-sensitive nanosensors and commercially available fluorescent nanoparticles (see supporting information), as well as the fluorescence response of particles spread across the whole area of the MEMS micro-hotplate and dispersed individual nanosensors.

A DeltaVision Elite (Applied Precision) with Olympus IX71 stand inverted microscope coupled with an Olympus U-Plan S-Apo 100 × 1.40 NA (oil, refractive index 1.520) objectives were used to visualise temperature-sensitive nanosensors dispersed on a glass coverslip, to show a representative high resolution distribution of nanosensors on a MEMS device surface. A CoolSNAPHQ^2^ charged coupled device camera (6.45 × 6.45 μm) pixel cell, 1000 kHz), interfaced Resolve3D softWoRx Acquire (version 5.5.0) software was used to acquire images (1024 × 1024, pixel size 0.06431 × 0.06431 × 0.500 μm). An InsightSSI solid state ﬂuorescence light source was used to excite red ﬂuorescence at 542/27 nm (85 mW), while collecting emitted ﬂuorescence at 594/45 nm. Transmission intensity and exposure times used were 50% and 0.10 s, respectively. The theoretical resolution of this optical system, calculated using the Rayleigh criterion, is 250 nm.

### 3.6 Control and characterisation of MEMS micro-hotplate

The MEMS micro-hotplate was electrically controlled using a Thurlby Thandar Instrument (PL303) power supply. The voltage and current to the device were monitored using an Agilent (34401A) multi-meter. The electrical temperature sensor of the micro-hotplate was characterised using a Signatone QuieTemp (S-1060R) hot chuck (±1 °C absolute accuracy). The electrical resistance was monitored using a Keithley (2400) source meter. Thermal simulations of the micro-hotplate were performed using the finite element method (FEM) with ANSYS (8.0). Thermal conductivity was modelled, by taking air convection into account, and the following material parameters; Tungsten 177 W/mK, Silicon Oxide 1.4 W/mK, Silicon Nitride 2.2 W/mK and Silicon 150 W/mK (25 °C).

## 4 Description of the MEMS micro-hotplate

**Fig. 1:**
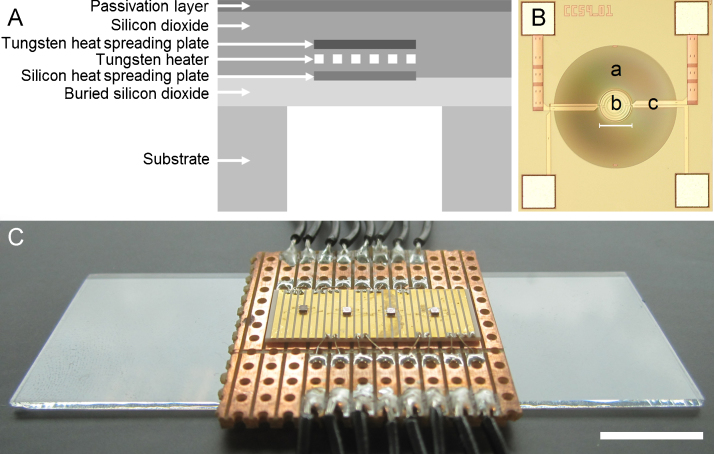
(A) Cross-section of the CMOS MEMS micro-hotplate. (B) Reflected light image of the silicon wafer of the MEMS device (scale bar = 150 μm), where *a*, *b* and *c* refer to the silicon dioxide membrane, tungsten heater and tungsten track, respectively. (C) Image of MEMS devices mounted on a circuit board (scale bar = 1 cm).

The MEMS micro-hotplate was developed by Cambridge CMOS Sensors Ltd and used as a tool to characterize the fluorescence response of temperature-sensitive nanosensors. The device consists of a multi-ring shaped resistive heater element, fabricated using CMOS tungsten metallization, embedded in a silicon dioxide membrane, Fig. 1A. Tungsten metallisation is ideally suited for micro-hotplates due to its high melting point and mechanical strength [35]. A resistive temperature sensing element is embedded within the CMOS oxide layers and both the sensor and the membrane have a silicon nitride passivation layer on top of them. The membrane was formed by back-etching using a post-CMOS deep reactive ion etch (DRIE) at a MEMS foundry. Unlike wet anisotropic etching, deep reactive ion etching allows easy formation of a circular membrane which has better mechanical stability because there are no sharp corners where stresses are concentrated. The metalized heater track is 4 μm wide. It has a diameter of 150 μm, covering a circular area of 0.017 mm^2^, and is thermally isolated in the centre of a 560 μm diameter membrane, Fig. 1B. For this study four MEMS devices of identical design were epoxied and wire bonded to a metalized ceramic circuit board from which electrical connections were made to a power supply and the multi-meter. The whole assembly is small enough to be mounted in the slide holder of a confocal microscope, Fig. 1C.

**Fig. 2:**
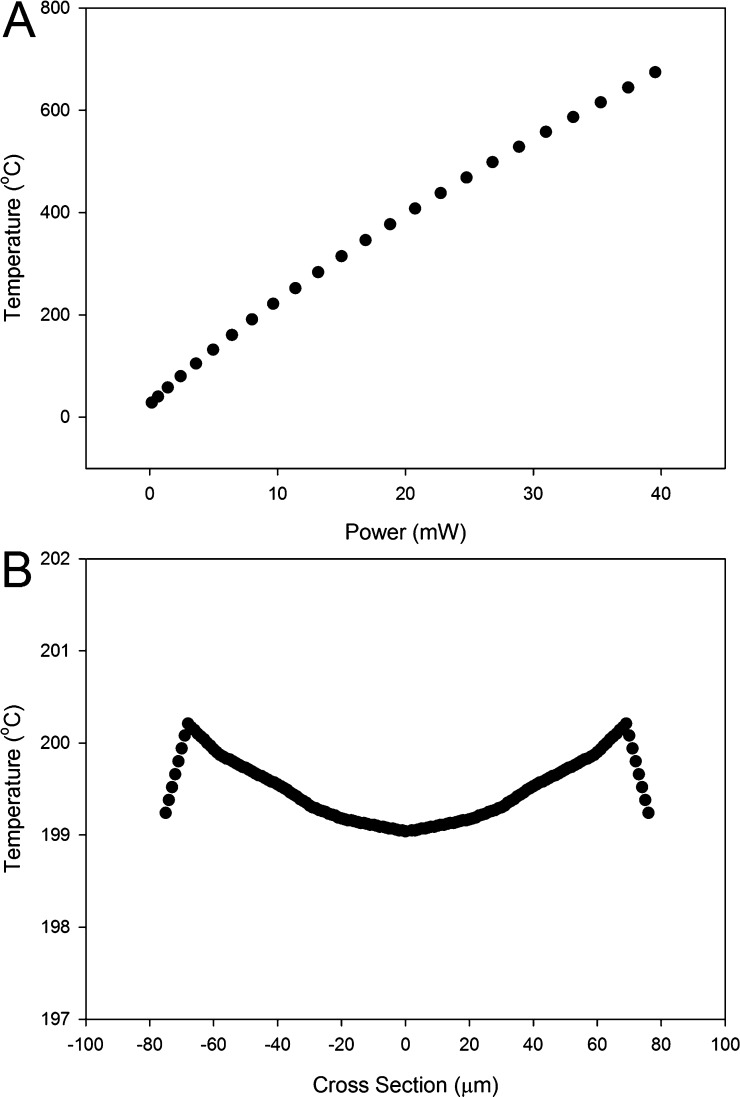
(A) Temperature rise of the MEMS micro-hotplate measured using the embedded resistive sensor at different electrical power levels. (B) Simulated temperature distribution across the surface of the MEMS micro-hotplate at 200 ̊C.

The MEMS micro-hotplate's electrical resistance at 25 °C is 60 Ω. To further describe the temperature dependent resistance, the following expression was used:

$$R=(R_{0}-R_{T})\times (1+\alpha (T-T_{0})+\beta {\left(T-T_{0}\right)}^{2})+R_{T}$$

where *R* and *R*_*T*_ are the micro-hotplate and track resistances, at elevated temperature (*T*) and 25 °C (*T*_0_), *α* and *β* are the temperature coefficients of resistance (TCR), which were experimentally determined to be 2.05 × 10^−3^ Ω/°C and 0.30 × 10^−6^ Ω/°C, respectively. The micro-hotplate is highly efficient, due to the thermal insulation provided by the silicone dioxide membrane surrounding the heater, and achieves an operating temperature of 200 °C at a power level of only 8.67 mW, Fig. 2A. Thermal simulations of the micro-hotplate at this temperature also indicate relatively high temperature uniformity (temperatures are within 99.5% of the peak), with ≤1.1 °C difference across the surface of the micro hot-plate, Fig. 2B. This high degree of temperature uniformity was engineered into the CMOS MEMS device through design the multi-ringed heat spreading hotplate.

## 5 Results and discussion

### 5.1 Development and characterization of temperature-sensitive nanosensors

**Scheme 1:**
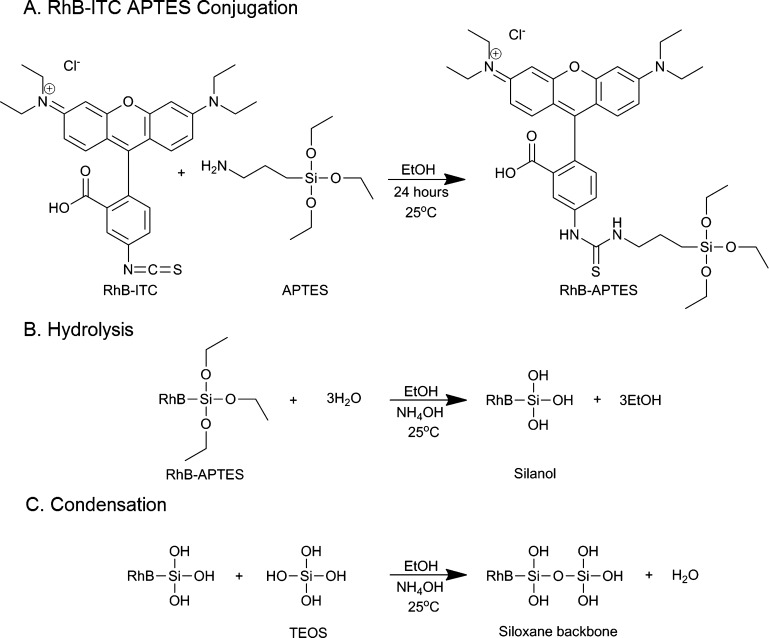
(A) Reaction scheme for rhodamine B isothiocyanate (RhB-ITC) and (3-aminopropyl) triethoxysilane (APTES) conjugation, (B) hydrolysis reaction between silica monomers and (C) condensation reaction of hydrolysed silica monomers.

Temperature-sensitive silica sol–gel nanosensors are spherical probes, composed of orthosilicate substituted monomers, tetraethylorthosilicate (TEOS) and the transducer RhB (3-aminopropyl)-triethoxysilane (APTES) conjugate. The RhB-APTES conjugate was formed through a nucleophilic addition reaction between RhB isothiocyanate (ITC) and APTES, Scheme 1A.

The monomers undergo a two-step hydrolysis and condensation reaction to produce a three-dimensional matrix [36]. During the hydrolysis reaction alkoxide groups are substituted with hydroxyl groups, forming silanols, Scheme 1B. Condensation of silanol groups forms the backbone of the silica matrix, the siloxane bond, Scheme 1C. Covalent linkage of RhB to the silica sol–gel matrix prevents the leaching of fluorophore, when in suspension, and possible deviation in fluorescent signal [37], [38].

**Fig. 3:**
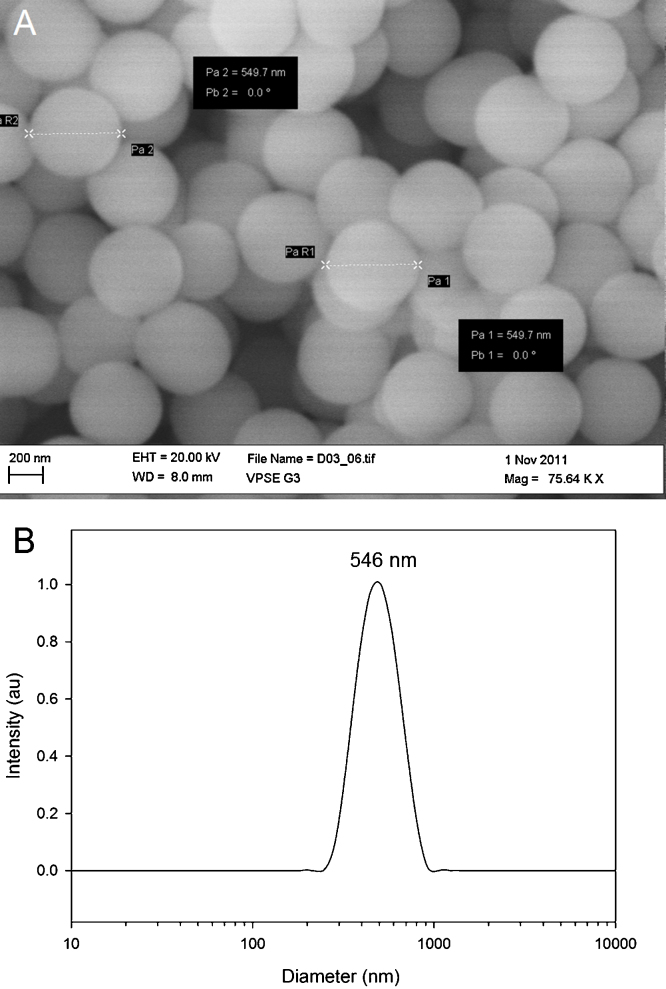
(A) Scanning electron microscopy (SEM) image of temperature-sensitive silica sol-gel nanosensors, with an average nanoparticle diameter of 529 ± 42 nm (*n* = 50). (B) Dynamic light scattering intensity distribution for silica sol–gel nanoparticles, showing nanoparticle diameters ranging between 300 nm and 1000 nm and an intensity distribution centered at 546 nm (PDI 0.048).

Nanoparticles with diameters ranging between 300 nm and 1000 nm were synthesized, so that they could be visualised using fluorescence confocal microscopy. Scanning electron microscopy (SEM) images show mono-dispersed nanoparticles, with a diameter of 529 ± 42 nm, Fig. 3A. These measurements were validated by dynamic light scattering, with an intensity distribution centered at 546 nm diameter (PDI = 0.048), Fig. 3B.

### 5.2 Fluorescence response of temperature-sensitive nanosensors

**Fig. 4:**
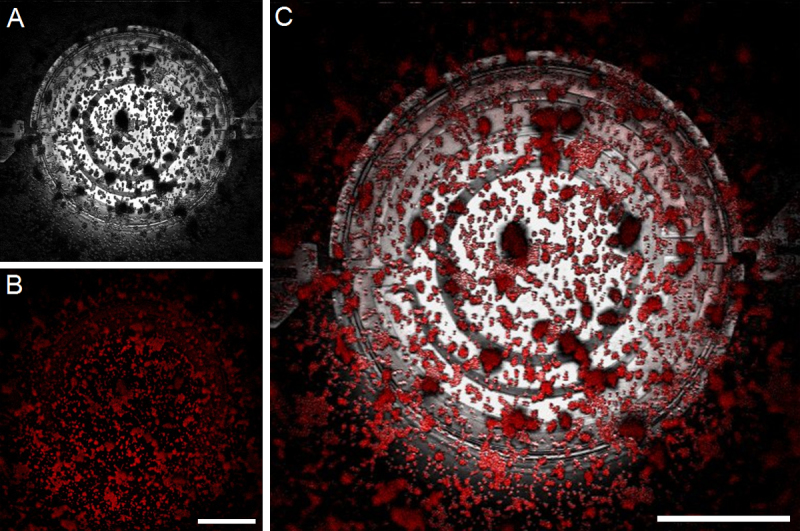
(A) Reflected light, (B) fluorescence and (C) merged channels of temperature-sensitive nanosensors deposited on MEMS micro-hotplate heater, imaged at 25 ̊C using a 50 × 0.80 NA (air) objective. Scale bar = 50 μm.

RhB based temperature-sensitive nanosensors have peak excitation and emission wavelengths at 554 nm and 573 nm, respectively [39]. Fig. 4 shows reflected light (Fig. 4A), fluorescence (Fig. 4B) and merged channel (Fig. 4C) image of dry temperature-sensitive nanosensors dispersed on the surface of the MEMS micro-hotplate, imaged at 25 °C using a 50 × 0.80 NA air objective. This air objective was selected to minimize the effects of changes in refractive index, as a result of changes to the thermal environment, maximizing the accuracy of particle size and fluorescence intensity measurements. Application of the Rayleigh criterion, as described by Hess *et al*. [40], using this objective lens and a Krypton 568 nm excitation laser, enabled calculation of the theoretical resolution limit of our optical system to be 442 nm. Therefore, the 50 × 0.80 NA air objective could be used to resolve the 550 nm temperature-sensitive nanosensors. Full width half maximum (FWHM) values, the width across the center of a nanoparticle at half its maximum intensity [41], were derived from a single in focus plane to compare the size of the nanosensors imaged using fluorescence microscopy. FWHM values derived for temperature-sensitive nanosensors were found to be comparable with commercially available fluorescent nanoparticles (see supporting information, Figs. S3 and S4).

**Fig. 5:**
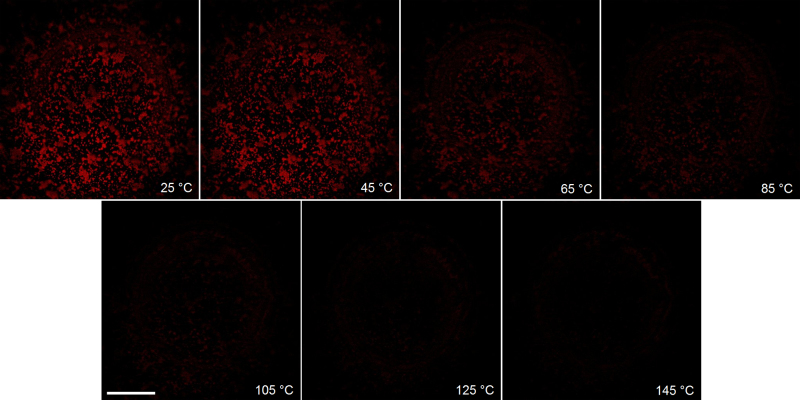
Fluorescence images of temperature-sensitive nanosensors, dispersed on the surface of MEMS micro-hotplate, between 25 ̊C and 145 ̊C. Scale bar = 50 μm.

**Fig. 6:**
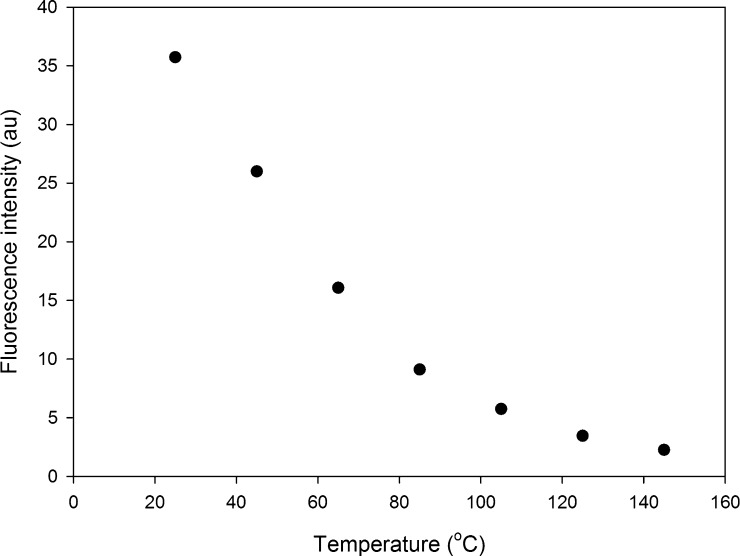
Fluorescence response of temperature-sensitive nanosensors, dispersed across the MEMS micro-hotplate surface, between 25 ̊C and 145 ̊C.

The fluorescence response of temperature-sensitive nanosensors decreases as the temperature of the MEMS micro-hotplate is increased from 25 °C to 145 °C, Fig. 5. Over this temperature range the fluorescence response of all nanosensors, dispersed over the whole surface of the MEMS micro-hotplate, falls in an exponential manner by 94% at 145 °C, Fig. 6. This fluorescence response was found to be reversible when cycling the temperature from high to low (see supporting information, Fig. S2).

**Fig. 7:**
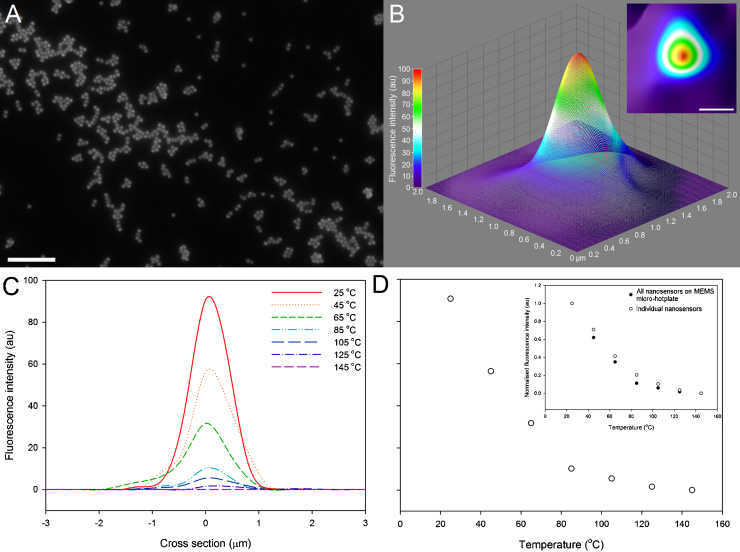
(A) High resolution image of dispersed temperature-sensitive nanosensors (scale bar = 5 μm). (B) Three and (B *inset*) two dimensional surface plot intensity plots of nanosensors, imaged at 25 ̊C using confocal microscopy (scale bar = 500 nm). (C) Line profile analysis of temperature-sensitive nanosensors, (FWHM = 0.973 ± 0.15 μm, 25 ̊C) and (D) peak fluorescence response of nanosensors, for temperatures ranging from 25 ̊C to 145 ̊C. (D *inset*) Comparison of peak temperature dependent fluorescence response from all nanosensors dispersed over the MEMS micro-hotplate surface and individual nanosensors.

High-resolution fluorescence images of temperature-sensitive nanosensors show spherical mono-size dispersed particles present, either as clusters or individual nanosensors, Fig. 7A. Three-dimensional surface intensity plots, Fig. 7B, can be generated for individual nanosensors imaged on the surface of the MEMS micro-hotplate, Fig. 7B *inset*. The distribution of fluorescence from the surface intensity plot is related to the number of fluorophores present at the centre and the edge of a spherical particle when imaged as a single in focus plane, rather than a change in temperature [42].

When a line profile is placed at the center of the surface intensity plot the temperature dependent fluorescence response of individual nanosensors can be determined for a range of temperatures, Fig. 7C. Fig. 7D shows the peak fluorescence intensity of individual particles decreases with increases in temperature of the MEMS micro-hotplate. The absolute fluorescence intensity from the nanosensors dispersed across the micro-hotplate surface, Fig. 6, is different from the fluorescence intensity measurements from individual nanosensors, Fig. 7D, due to the absence of fluorescence from areas not containing any nanosensors. However, when the fluorescence intensity is normalized the response of the dispersed nanosensors across the surface of the micro-hotplate is not statistically different to the fluorescence response from individual nanosensors (*p* < 0.05), Fig. 7D *inset*. At 145 °C the fluorescence response of individually imaged temperature-sensitive nanosensors appears to be fully quenched as the nanosensors do not exhibit a detectable fluorescence signal. Therefore, this temperature could be considered as temperature detection limit of RhB based silica sol–gel temperature-sensitive nanosensors.

## 6 Conclusion

In conclusion, we have demonstrated the application of a custom designed CMOS MEMS device for the thermo-optical characterization of an RhB based temperature-sensitive nanosensor. The MEMS device is a low power, high temperature, micro-hotplate which is small enough to be mounted on a conventional slide holder of a microscope and can be manufactured using low cost, high volume, CMOS fabrication processes. Simulations of the temperature profile of the whole surface of the micro-hotplate show 99.5% temperature uniformity at 200 °C. Application of the Rayleigh criterion confirmed individual nanosensors, approximately 550 nm in diameter, could be resolved using our optical imaging system. The temperature dependent fluorescence intensity of nanosensors dispersed across of the surface of the micro-hotplate, demonstrated a 94% drop in fluorescence intensity when the temperature was increased from 25 to 145 °C. Individual nanosensors did not exhibit any observable fluorescence at 145 °C and this temperature was taken to be the detection limit of the nanosensors. Temperature-sensitive silica sol–gel nanosensors are simple and inexpensive to manufacture and may find potential applications in the thermal characterization of nano/micro scale electronic, mechanical and biological systems. Through modification of the nanosensor synthesis protocol [43] and the introduction of super-resolution fluorescence microscopy [44], we believe temperature mapping could be achieved, using sub 100 nm temperature-sensitive nanosensors.

## References

[1] Wang X., Wolfbeis O., Meier R. (2013). Luminescent probes and sensors for temperature. Chemical Society Reviews.

[2] Oyama K., Takabayashi M., Takei Y., Arai S., Takeoka S., Ishiwata S., Suzuki M. (2012). Walking nanothermometers spatiotemporal temperature measurement of transported acidic organelles in single living cells. Lab on a Chip.

[3] Meyer C.W., Meier D.C., Montgomery C.B., Semancik S. (2006). Temperature measurements of microhotplates using fluorescence thermometry. Sensors and Actuators A: Physical.

[4] Gota C., Okabe K., Funatsu T., Harada Y., Uchiyama S. (2009). Nanogel thermometer for intracellular thermometry. Journal of the American Chemical Society.

[5] Li S., Zhang K., Yang J.M., Lin L.W., Yang H. (2007). Single quantum dots as local temperature markers. Nano Letters.

[6] Graham E.M., Iwai K., Uchiyama S., de Silva A.P., Magennis S.W., Jones A.C. (2010). Quantitative mapping of aqueous microfluidic temperature with sub-degree resolution using fluorescence lifetime imaging microscopy. Lab on a Chip.

[7] Low P., Kim B., Takama N., Bergaud C. (2008). High-spatial-resolution surface-temperature mapping using fluorescent thermometry. Small.

[8] Baffou G., Kreuzer M.P., Kulzer F., Quidant R. (2009). Temperature mapping near plasmonic nanostructures using fluorescence polarization anisotropy. Optics Express.

[9] Baffou G., Girard C., Quidant R. (2010). Mapping heat origin in plasmonic structures. Physical Review Letters.

[10] Vitez I.M., Newman A.W., Davidovich M., Kiesnowski C. (1998). The evolution of hot-stage microscopy to aid solid-state characterizations of pharmaceutical solids. Thermochimica Acta.

[11] Boccaccini A.R., Hamann B. (1999). In situ high-temperature optical microscopy. Journal of Materials Science.

[12] Arata H.F., Low P., Ishizuka K., Bergaud C., Kim B., Noji H., Fujita H. (2006). Temperature distribution measurement on microfabricated thermodevice for single biomolecular observation using fluorescent dye. Sensors and Actuators B: Chemical.

[13] Kamino T., Saka H. (1993). A newly developed high-resolution hot stage and its application to materials characterization. Microscopy Microanalysis Microstructures.

[14] Fung S.K.H., Tang Z.N., Chan P.C.H., Sin J.K.O., Cheung P.W., Thermal Analysis (1996). Design of a micro-hotplate for integrated gas-sensor applications. Sensors and Actuators A: Physical.

[15] Tsamis C., Nassiopoulou A.G., Tserepi A. (2003). Thermal properties of suspended porous silicon micro-hotplates for sensor applications. Sensors and Actuators B: Chemical.

[16] Ali S.Z., Santra S., Haneef I., Schwandt C., Kumar R.V., Milne W.I., Udrea F., Guha P.K., Covington J.A., Gardner J.W., Garofalo V. (2009). Nanowire hydrogen gas sensor employing CMOS micro-hotplate. IEEE Sensors.

[17] Haneef I., Burzo M., Ali S.Z., Komarov P., Udrea F., Raad P.E. (2010). Thermal characterization of SOI CMOS micro hot-plate gas sensors. 16th International Workshop on Thermal Investigations of ICs and Systems (THERMINIC).

[18] Ali S.Z. (2007). Electro-Thermo-Mechanical Study of Membrane Devices for Smart IC Technologies. http://www.warwick.ac.uk/fac/sci/eng/research/sensors/mbl/database/pphd/sali.pdf.

[19] Santra S., Udrea F., Guha P.K., Ali S.Z., Haneef I. (2010). Ultra-high temperature (>300̊ C) suspended thermodiode in SOI CMOS technology. Microelectronics Journal.

[20] Ali S.Z., Udrea F., Milne W.I., Gardner J.W. (2008). Tungsten-based SOI microhotplates for smart gas sensors. Journal of Microelectromechanical Systems.

[21] Wang B., Qiao L. (2011). A MEMS differential scanning calorimeter for thermodynamic characterization of biomolecules. IEEE 24th International Conference on Micro Electro Mechanical Systems (MEMS).

[22] Zhang S., Rabin Y., Yang Y., Asheghi M. (2007). Nanoscale calorimetry using a suspended bridge configuration. Journal of Microelectromechanical Systems.

[23] Efremov M.Y., Olson E.A., Zhang M., Zhang Z., Allen L.H. (2003). Glass transition in ultrathin polymer films: calorimetric study. Physical Review Letters.

[24] Aceros J.C., McGruer N.E., Adams G.G. (2008). Microelectromechanical system microhotplates for reliability testing of thin films and nanowires. Journal of Vacuum Science and Technology B.

[25] Low P., Takama N., Kim B., Bergaud C. (2007). Using dried rhodamine B fluorescence for temperature characterization of sub-micron scale devices. IEEE International Solid-State Sensors, Actuators and Microsystems Conference.

[26] Ferguson J., Mau A.W.H. (1973). Spontaneous and stimulated emission from dyes—spectroscopy of neutral molecules of acridine-orange, proflavine, and rhodamine-B. Australian Journal of Chemistry.

[27] Glawdel T., Almutairi Z., Wang S., Ren C. (2009). Photobleaching absorbed rhodamine B to improve temperature measurements In PDMS microchannels. Lab on a Chip.

[28] Alford R., Simpson H.M., Duberman J., Hill G.C., Ogawa M., Regino C., Kobayashi H., Choyke P.L. (2009). Toxicity of organic fluorophores used in molecular imaging: literature review. Molecular Imaging.

[29] Pittman J.L., Henry C.S., Gilman S.D. (2003). Experimental studies of electroosmotic flow dynamics in microfabricated devices during current monitoring experiments. Analytical Chemistry.

[30] Duong H.D., Rhee J.I. (2007). Exploitation of thermo-effect of rhodamine B entrapped in sol–gel matrix and silica gel for temperature detection. Sensors and Actuators B: Chemical.

[31] Ross D., Gaitan M., Locascio L.E. (2001). Temperature measurement in microfluidic systems using a temperature-dependent fluorescent dye. Analytical Chemistry.

[32] Kim K., Lee Y.M., Lee J.W., Shin K.S. (2009). Metal-enhanced fluorescence of rhodamine B isothiocyanate from micrometer-sized silver powders. Langmuir.

[33] Peng H.-S., Huang S.-H., Wolfbeis O.S. (2010). Ratiometric fluorescent nanoparticles for sensing temperature. Journal of Nanoparticle Research.

[34] Soraru G.D., Suttor D. (1999). High temperature stability of sol–gel-derived SiOC glasses. Journal of Sol–Gel Science and Technology.

[35] Chen L.Y., Santos E.J.P., Macdonald N.C. (1993). An isolation technology for joined tungsten MEMS IEEE. Micro Electro Mechanical Systems, Proceedings: An Investigation of Micro Structures, Sensors, Actuators, Machines, and Systems.

[36] Stöber W., Fink A., Bohn E. (1968). Controlled growth of monodisperse silica spheres in micron size range. Journal of Colloid and Interface Science.

[37] Doussineau T., Smaihi M., Mohr G.J. (2009). Two-dye core/shell zeolite nanoparticles: a new tool for ratiometric pH measurements. Advanced Functional Materials.

[38] Chauhan V.M., Orsi G., Brown A., Pritchard D.I., Aylott J.W. (2013). Mapping the pharyngeal and intestinal pH of *Caenorhabditis elegans* and real-time luminal pH oscillations using extended dynamic range pH-sensitive nanosensors. ACS Nano.

[39] Shah J.J., Gaitan M., Geist J. (2009). Generalized temperature measurement equations for rhodamine B dye solution and its application to microfluidics. Analytical Chemistry.

[40] Hess S.T., Girirajan T.P.K., Mason M.D. (2006). Ultra-high resolution imaging by fluorescence photoactivation localization microscopy. Biophysical Journal.

[41] Stelzer E.H.K. (1998). Contrast, resolution, pixelation, dynamic range and signal-to-noise ratio fundamental limits to resolution in fluorescence light microscopy. Journal of Microscopy.

[42] Waters J.C. (2009). Accuracy and precision in quantitative fluorescence microscopy. Journal of Cell Biology.

[43] Ma K., Werner-Zwanziger U., Zwanziger J., Wiesner U. (2013). Controlling growth of ultrasmall sub 10 nm fluorescent mesoporous silica nanoparticles. Chemistry of Materials.

[44] Huang B., Bates M., Zhuang X.W. (2009). Super-resolution fluorescence microscopy. Annual Review of Biochemistry.

