# Supplementary material for: Thermo-optical characterization of fluorescent rhodamine B based temperature-sensitive nanosensors using a CMOS MEMS micro-hotplate
Source: Sens Actuators B Chem. 2014 Mar 1;192:126–33. doi: 10.1016/j.snb.2013.10.042 (PMC4376176; doi:10.1016/j.snb.2013.10.042)
Supplement: Supplementary file 1 [file mmc1.pdf]

## Supporting Information

# Thermo-optical characterization of fluorescent rhodamine B based temperature-sensitive nanosensors using a CMOS MEMS micro-hotplate

*Veeren M Chauhan,[a] Richard H Hopper,[b] Syed Z Ali,[b] Emma M King,[c] Florin Udrea,[b,d] Chris H Oxley,[e] and Jonathan W Aylott[a]**

[a]Laboratory of Biophysics and Surface Analysis, School of Pharmacy, University of Nottingham, Boots Science Building, University Park, Nottingham NG7 2RD, UK

[b]Cambridge CMOS Sensors, Suite 820, 2nd Floor, St Andrews House, 59 St Andrews Street, Cambridge CB2 3BZ, UK

[c]Advanced Microscopy Unit, School of Biomedical Sciences, University of Nottingham, Queens Medical Centre, Nottingham NG7 2UH, UK

[d]Electrical Engineering Division, Engineering Department, University of Cambridge, 9 JJ Thomson Avenue, Cambridge CB3 0FA, UK

[e]Engineering, Faculty of Technology, De Montfort University, Queens Building, The Gateway, Leicester LE1 9BH, UK

**Supporting Methods**

*Movement of MEMS Hotplate with Changes in Temperature:* The MEMS hotplate was placed in a fluorescence confocal microscope, and was imaged using a 50x 0.80 NA (air) objective. A saturation lookup table was initially visualised the centre of the hot plate at 25 °C, such that the centre plate was completely saturated. The temperature of the hot plate was gradually increased to 605 °C, in 20 °C steps. The change in µm of the focal plane was recorded at each temperature ($n$ = 3).

*Temperature cycling:* Nanoparticles were suspended in deionised water (50 µL, 1mg/mL) and deposited and dried of the surface of a quartz heater plate of a Linkam Scientific DSC600 stage. For thermal cycles samples were heated at a rate of 30 °C/min, controlled by Linksys 32 software, to well-defined temperature points between 0 and 200°C and allowed to thermally stabilize for 2 minutes after attaining each new temperature.

*Environmental Scanning Electron Microscopy (ESEM):* TetraSpeck 500 nm (TS500) nanoparticles (Invitrogen™, Paisley, United Kingdom) were spread and dried on 400 mm$^2$ mica placed on an aluminium scanning electron microscope stub (Agar Scientific). The sample was platinum coated (90 seconds, 2.2 kV, 20 mA plasma current and 4x10$^{-2}$ mbar vacuum) using a Polaron SC7640 sputter coater. A Philips XL30 ESEM-FEG scanning electron microscope was used to image dry nanoparticles (20 kV, 10.1 mm working distance). The measured diameters were validated using dynamic light scattering.

*Dynamic light scattering:* Dynamic light scattering was performed using a Viscotek (802) system. The system is equipped with a 50 mW laser source (830 nm), operating at an angle of 90°. TS500 nanoparticles (10 µL) were suspended in 1 mL of deionised water. Measurements (10 runs, 25 °C) were made using a Hellma® Analytics quartz cuvette (1.5 mm diameter). The mean hydrodynamic diameter of the particles was computed from the intensity of the scattered light using the OmniSize 3.0 software.

*Full width half max (FWHM) analysis of TetraSpeck 500 nm (TS500) nanoparticles*: TS500 nanoparticles (5 µL) were spread on the surface of a glass slide and allowed to dry. A Leica SP2 confocal fluorescence microscope coupled with a Leica HC PL FLUOTAR 50x 0.8 NA (air) (pixel size 0.39 x 0.39 µm, pinhole 0.119 mm (1 Airy)) was used to image TS500 nanoparticles, at 25 °C. A He-Ne 568 nm laser was used as excitation source. A photomultiplier tube (1225 HV, 100 offset) was used to collect fluorescence between 580 nm and 625 nm. FWHM values were calculated by fitting a Gaussian distribution curve, which was solved for half the maximal intensities of a line profile of single TS500 nanoparticle ($n$ = 6). Student's $t$ test was used to identify significant differences FWHM of temperature-sensitive nanosensors and TS500 nanoparticles.

**Supporting Results**

*Movement of MEMS Hotplate with Changes in Temperature*: Fig. S1 shows the MEMS hotplate buckles by 9.39 ± 5.4 µm when the temperature is increased from 25 to 605 °C. By use of confocal microscope, rather than a conventional fluorescence microscope, the temperature sensitive nanoparticles deposited on the surface of the MEMS hotplate can be kept in focus.

*Temperature cycling:* Fig. S2 shows fluorescence intensity levels recorded during thermal cycling of the aggregated sensors from 200 °C to 0 °C. The response of the sensors was reversible, although some overall reduction in peak intensity of 18% was observed over the 70 minute time period of the experiment.

*Size characterisation of TetraSpeck 500 nm (TS500) nanoparticles:* ESEM of TS500 show nanoparticles with an average diameter of 485 ± 20 nm, Fig. S3A. These measurements were validated with DLS which show nanoparticles with diameters ranging from 300 to 1000 nm, with an intensity distribution centred at 531 nm, Fig. S3B.

*Full width half max (FWHM) analysis of TetraSpeck 500 nm (TS500) nanoparticles*: Analysis of TS500 nanoparticles shows particles with a FWHM of 1.04 ± 0.44 µm, Fig. S4. The FWHM of TS500 is not statistically different to temperature-sensitive nanoparticles ($p < 0.05$).

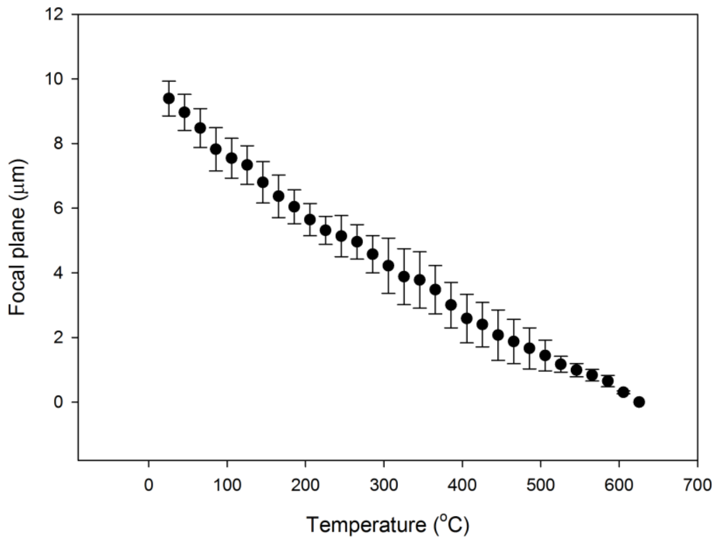


**Fig. S1.** Temperature induced changes in focal plane, as measured used saturation of reflected light from the surface of the MEMS hot plate, using confocal microscopy.

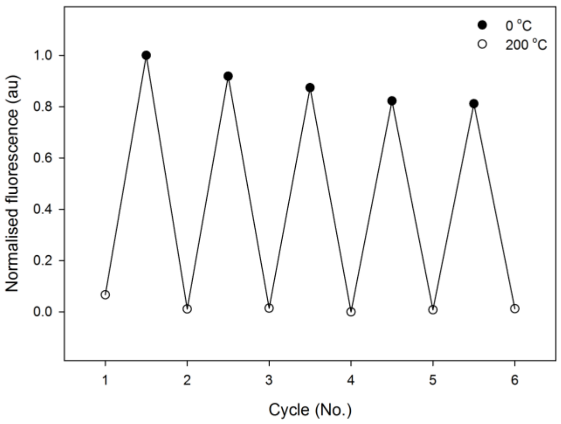


**Fig. S2**. Fluorescence intensity measured for temperature-sensitive nanosensors when cycled between 0 and 200 °C.

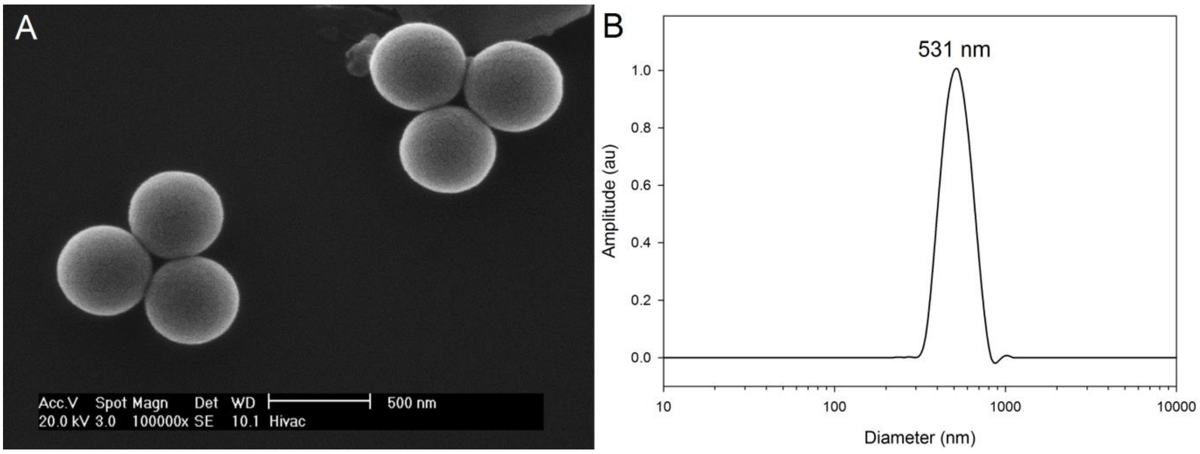


**Fig. S3**. (A) Environmental scanning electron microscopy image TetraSpeck 500 nm (TS500) nanoparticles, with an average nanoparticle diameter and (B) dynamic light scattering intensity distribution for TS500 nanoparticles.

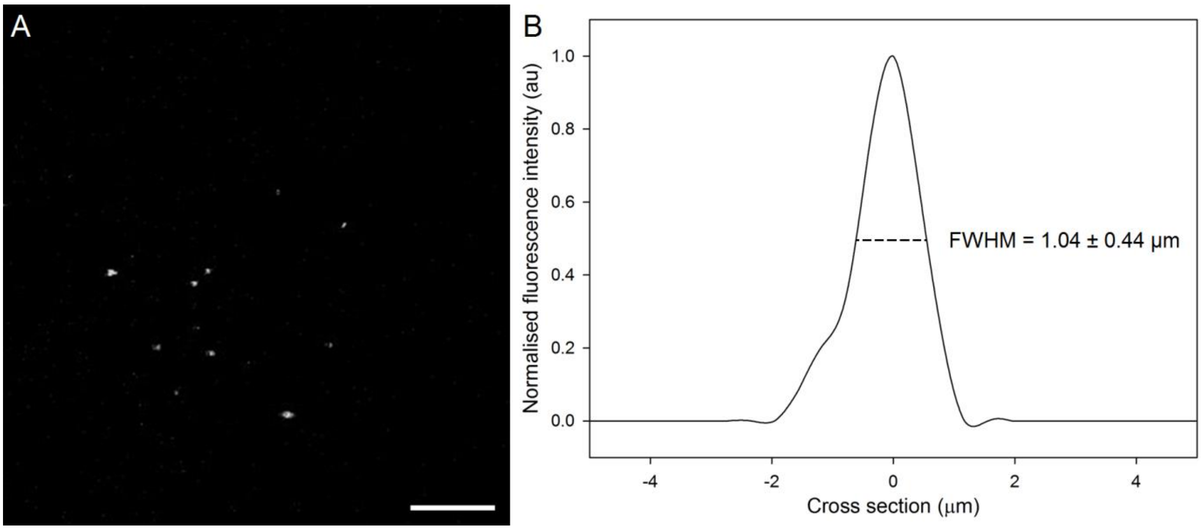


**Fig. S4.** (A) Image and (B) full width half max (FWHM) analysis of TetraSpeck 500 nm (TS500) nanoparticles ($n$ = 6).
